# Case Report: Retroperitoneal bronchogenic cyst of imaging features and pathological analysis in a young female

**DOI:** 10.3389/fmed.2025.1746605

**Published:** 2026-01-14

**Authors:** Wenhao Yuan, Siying Liu, Qingqi Ren, Erbao Chen, Zewei Lin

**Affiliations:** 1 Peking University Shenzhen Hospital, Shenzhen, Guangdong, China; 2 Shenzhen University, Shenzhen, Guangdong, China; 3 Department of Hepatobiliary and Pancreatic Surgery, Peking University Shenzhen Hospital, Shenzhen, Guangdong, China; 4 Guangdong Medical University, Zhanjiang, Guangdong, China

**Keywords:** bronchogenic cysts, diagnosis, imaging, pathology, retroperitoneum

## Abstract

Bronchogenic cysts are cystic lesions arising from the bronchial epithelium. They are very rare in the retroperitoneum. In this report, we present the case of a young woman found to have a cystic mass in the hepatorenal space during a routine health examination. Imaging studies confirmed a cystic lesion, and surgical excision revealed adherence to the lower esophagus and gastroesophageal junction. This retroperitoneal location, along with non-specific imaging features overlapping with other benign masses, poses a significant diagnostic challenge. The imaging appearance of bronchogenic cysts can be variable, complicating clinical diagnosis. We highlight the critical importance of correlating imaging with histopathological examination for accurate diagnosis. Although typically benign, potential complications and the risk of misdiagnosis warrant clinical vigilance. In summary, in this case, we tried to present clinical findings and management. We aimed to provide a foundation for future studies on the epidemiological characteristics of this disease.

## 1 Introduction

Bronchogenic cysts are cystic lesions that originate from the bronchial epithelium (1, 2). It usually originates in the thoracic cavity and is very rare. Bronchogenic cysts are more common in young males (3). However, recently, bronchogenic cysts in young females, particularly in the hepatorenal space in the retroperitoneum is more frequently, even though it is very rare (3). These cysts are incidentally detected during routine imaging examinations. Clinical presentation may be asymptomatic or present with respiratory symptoms. It may also be complicated by infections and compressive symptoms (4). Therefore, we should do more epidemiologic studies on bronchogenic cysts to understand their pathogenesis and clinical features.

The patient who presented in this case report had initially consulted the hospital due to the cystic mass discovered in the hepatorenal space on routine check-up (5). The patient had no apparent complaint before consultation. A cystic mass was detected on imaging examinations. Intraoperative exploration revealed that the mass was adherent to the lower esophagus and gastroesophageal junction. The mass was successfully removed. To our knowledge, the present case is unique due to the occurrence of cysts in the retroperitoneum. The imaging features of the mass were considered a benign soft tissue lesion, such as a lymphangioma, which increased the difficulty of initial diagnosis (4). The literature review revealed that imaging findings of bronchogenic cysts may present of various types, which confuse clinicians (5).

The clinical value of this case is that the occurrence of a bronchogenic cyst in the retroperitoneum was described in detail, and the integration of imaging and pathology should be considered. Although the lesion is benign, clinicians should be vigilant about possible complications and misdiagnosis of this disease (6). This case report could provide important references and guidance for similar cases. The treatment and diagnosis of this lesion could help clinicians understand this disease.

In conclusion, this case not only could provide important experiences on clinical presentation and surgical treatment for the readers about bronchogenic cyst, but also could provide a basis for further study on epidemiological characteristics and pathological studies of this disease. We expect that clinicians could improve their ability to diagnose when meeting similar cases by summarizing and analyzing similar cases.

## 2 Case presentation

**Figure 1:**
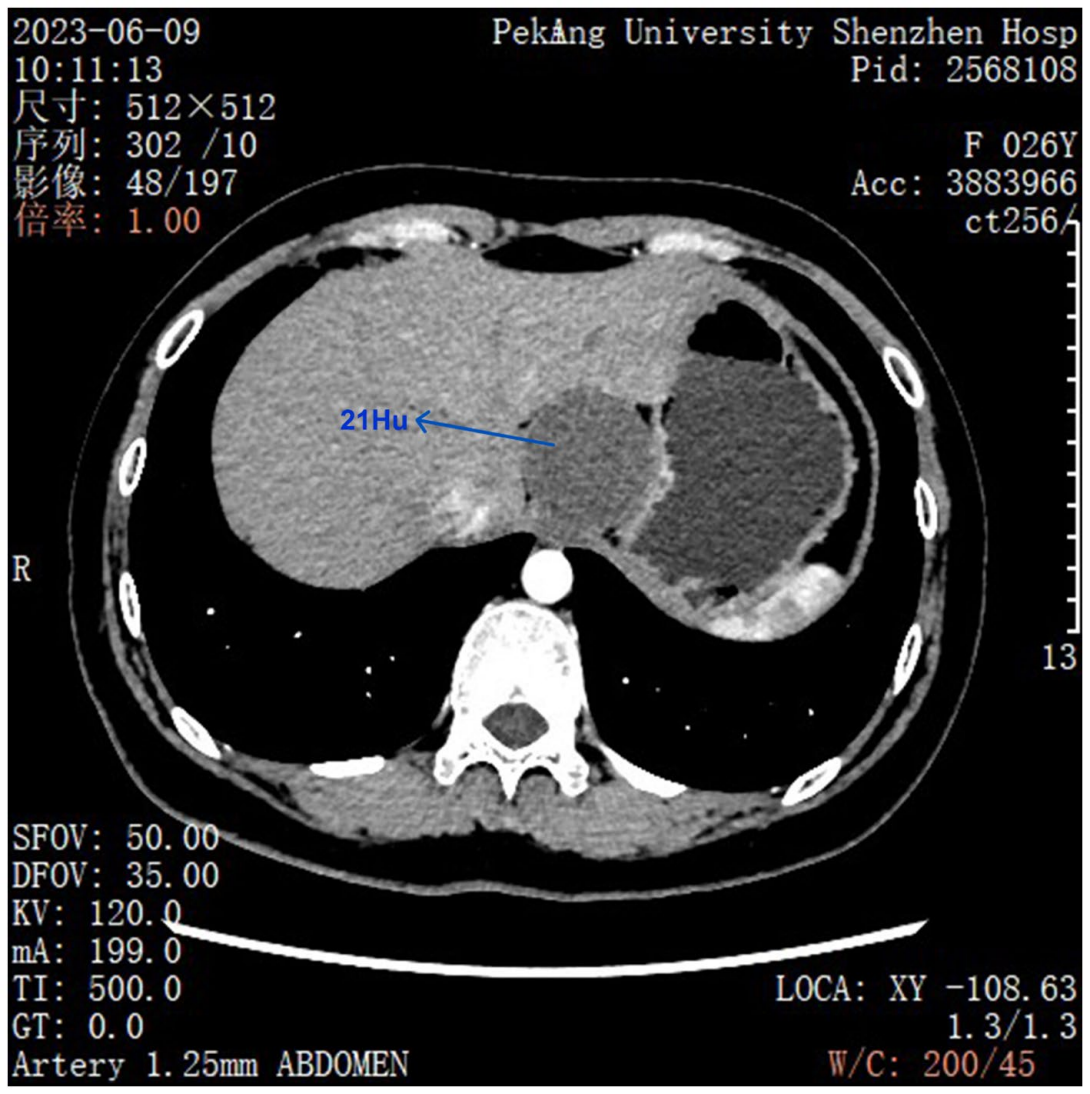
Preoperative CT scan revealed a quasi-circular low-density shadow in the hepatogastric space, approximately 48 mm*46 mm in size, with a CT value of approximately 21 HU. Slight calcification was visible at its edge. No significant enhancement was observed after contrast administration, while the boundary remained clear. Compression changes affected the adjacent left lobe of the liver and the gastric wall.

This is a case of a 26-year-old woman. Abdominal CT report at routine check-up in February 2023 described a cystic mass in the hepatorenal space measuring approximately 4.2 cm in diameter. Follow-up with the same patient after 3 months. Repeat abdominal enhanced CT demonstrated that the cystic mass in the hepatorenal space had enlarged, measuring approximately 4.8 cm × 4.6 cm in size. This is a benign lesion, with lymphangioma as a possible diagnosis (Figure 1).

## 3 Diagnostic assessment

**Figure 2:**
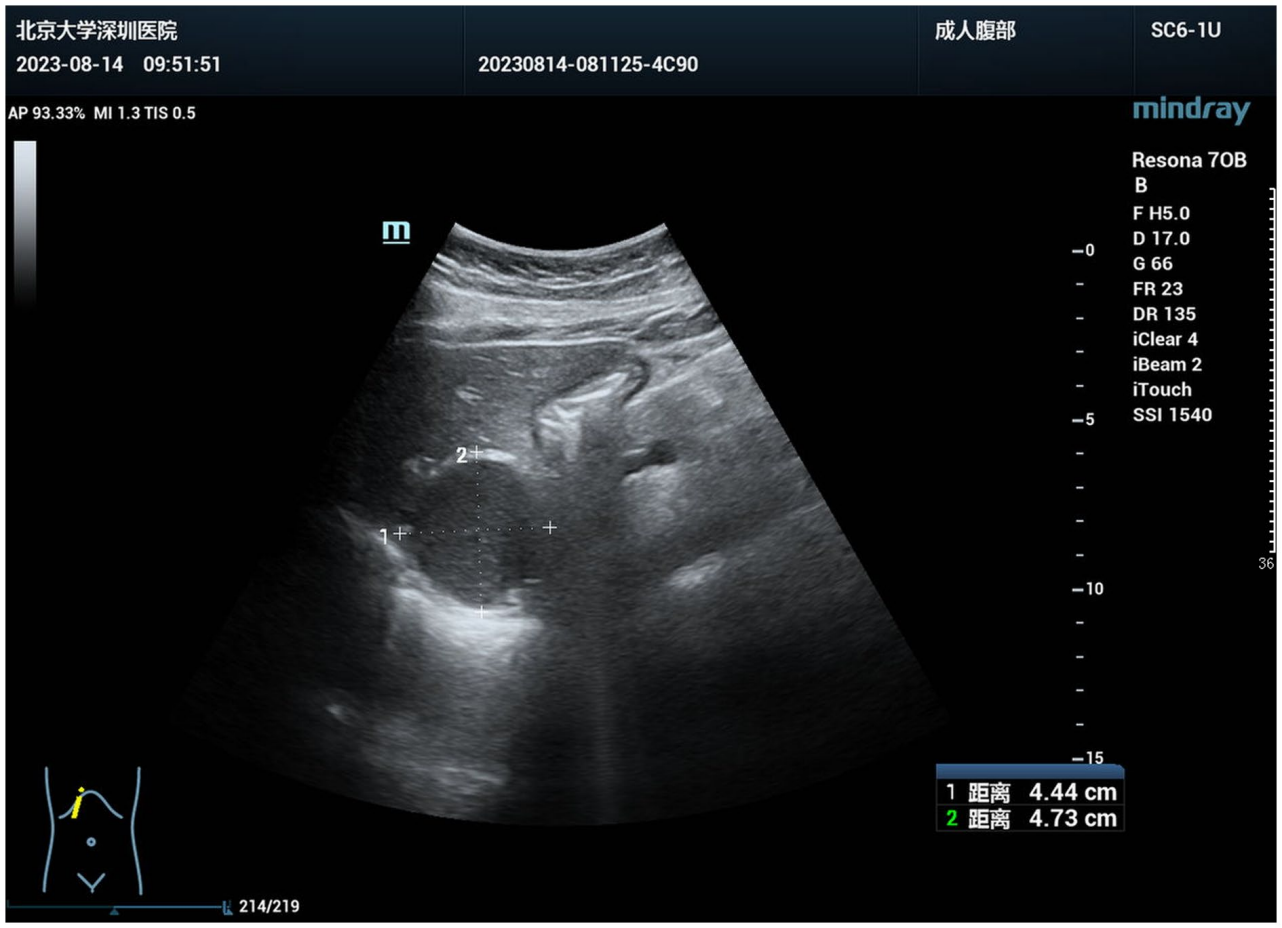
Preoperative color Doppler ultrasound revealed an anechoic mass in the hepatogastric space. The mass was well-defined, quasi-circular, with enhanced posterior echoes and poor internal echogenicity.

**Figure 3:**
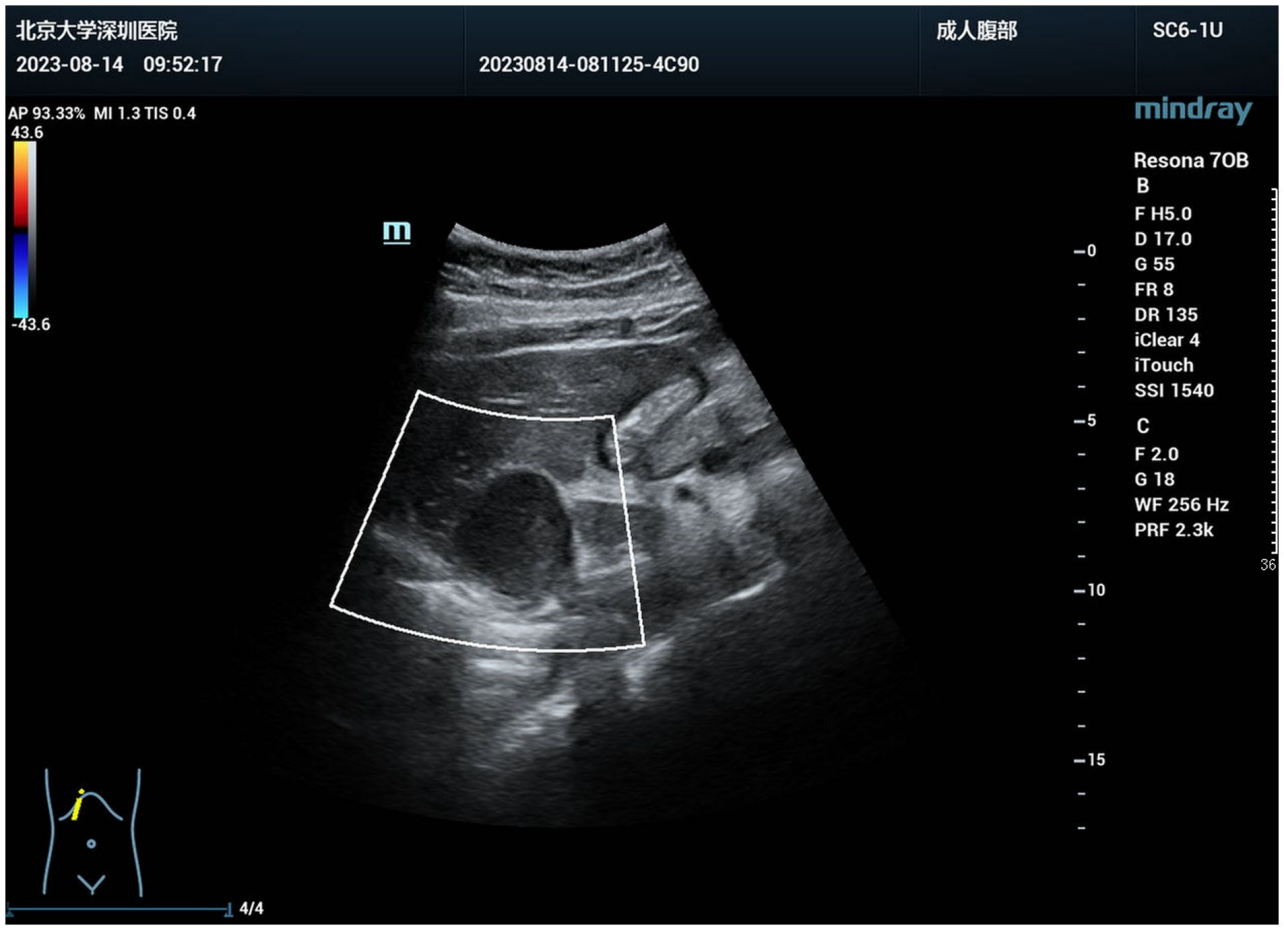
CDFI performed preoperatively revealed no significant blood flow signals within or around the tumor.

To further elucidate the nature of the lesion, the patient had an ultrasound scan. The scan made visible a well-defined mass in the space between the liver and stomach, roughly 4.7 cm × 4.4 cm in size. The mass presented as round, with well-defined boundaries, weak internal echogenicity, and heightened posterior echogenicity (Figure 2). Color Doppler flow imaging (CDFI) examination showed no significant blood flow signals either inside or around the mass (Figure 3). Based on the imaging results, the diagnosis was a retroperitoneal cystic mass with a high probability of being a benign lesion. Along with the imaging results, the diagnosis was a retroperitoneal cystic mass with a high chance of being a benign lesion. Due to various reasons, the MRI examination was not completed in this case during the preoperative check. The mass is often confused with lymphangioma, enteric duplication cyst, or adrenal lesions and requires differential diagnosis. Lymphangioma generally manifests itself as a cyst-like formation that is multilocular. In this instance, the lesion was unilocular and without septa, thus lymphangioma was less likely. In this case, enteric duplication cysts, which are normally located next to the digestive tracts, were not suitable for the position or shape of the lesion. Adrenal lesions, such as an adrenal cyst, were also put into consideration. On CT and ultrasound, the mass was, however, distinctly differentiated from the adrenal gland. Moreover, there were no clinical endocrine symptoms present, which allowed ruling out this diagnosis somewhat. The imaging features suggested a benign cystic lesion. These included a regular shape, well-defined margins, absence of internal blood flow, and posterior acoustic enhancement. From imaging and ultrasound findings, the patient got a final diagnosis of a benign retroperitoneal cystic mass. To authenticate the conjectured diagnosis, the patient was admitted on 15 August 2023 for presurgical assessments prior to the surgical procedure. To confirm this diagnosis, the patient was admitted on 15 August 2023, for pre-operative examinations before surgical treatment.

## 4 Treatment

The patient had the retroperitoneal mass removed laparoscopically under general anesthesia. Intraoperative exploration disclosed that the tumor was placed below the left lobe of the liver, near the level of the gastric cardia, measuring approximately 5 cm across. The upper and surrounding areas of the tumor had no binding, yet the bottom was tightly attached to the lower esophagus and gastroesophageal junction. The surgical team successfully detached the mass and sent it to pathology for a regular examination.

## 5 Outcomes and follow-up

**Figure 4:**
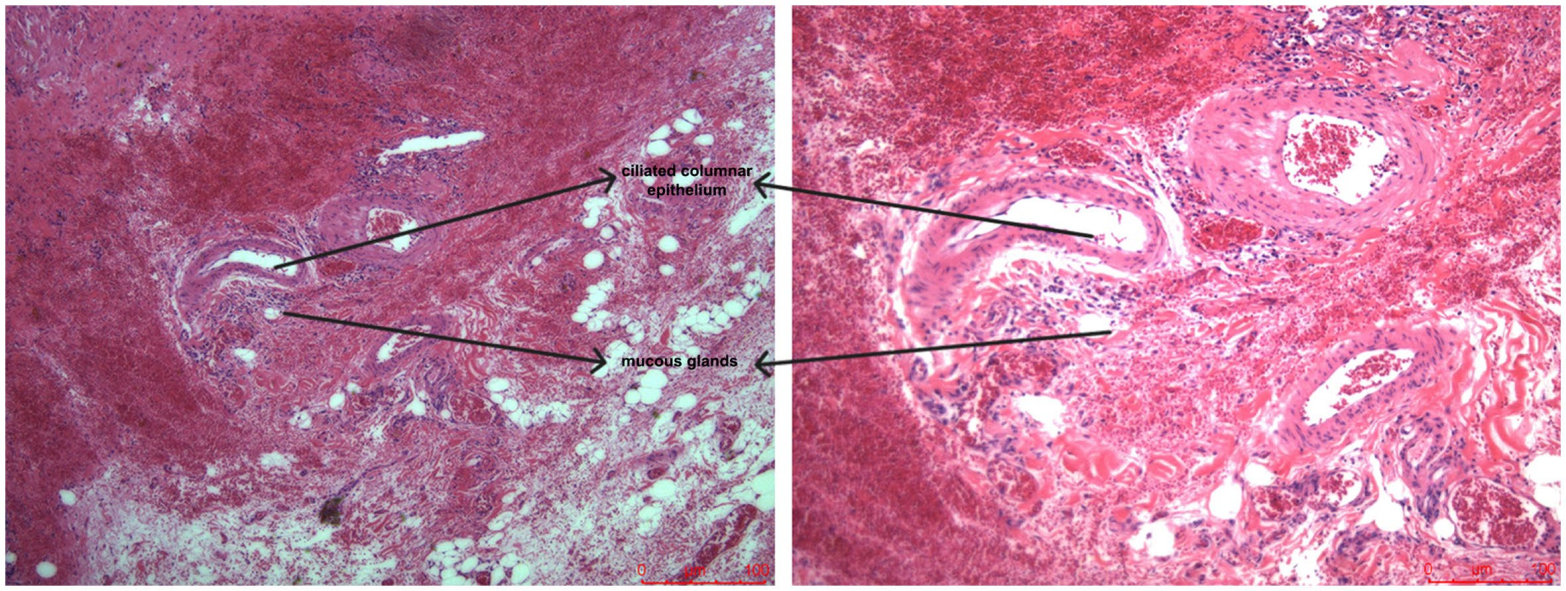
Postoperative pathology suggests that the tumor is cystic, containing mucinous material. The inner wall is lined with ciliated columnar epithelium, with areas of local epithelial loss. Multinucleated giant cell reaction, infiltration of histiocytes and inflammatory cells, and focal epithelial keratinization are also observed. Smooth muscle is seen in the cyst wall.

Post-operative pathology revealed that the mass was a bronchogenic gastric cyst (Figure 4). The patient recovered nicely after surgery and was safely discharged after the observation process. This case details the diagnosis and treatment of a bronchogenic retroperitoneal mass, providing insights for relevant inquiries.

## 6 Discussion

In this study, we reported a case of bronchogenic cyst in a young female. Bronchogenic cysts are very rare in young females. The most reported references believed that a bronchogenic cyst was accompanied by a bronchial developmental abnormality or incomplete differentiation (7). Most patients were male and manifested typical symptoms in childhood and adolescence (4). However, according to our experience, the average age of patients with bronchogenic cysts was 16.4 years old, and patients presented with respiratory symptoms (8). However, the imaging in our case showed a retroperitoneal mass, which was extremely rare, and might cause misdiagnosis with other benign masses, such as lymphangioma, enteric duplication cyst, or adrenal lesions (9). For instance, Hu et al. (10) reported three cases of retroperitoneal bronchogenic cysts clinically mimicking adrenal masses, emphasizing through a literature review that such cysts are often misinterpreted as lymphangiomas, enteric duplication cysts, or adrenal lesions due to overlapping imaging features. This aligns with our observation that differentiation from other benign masses is challenging without histopathological confirmation. Similarly, Xiang et al. (11) provided a comprehensive analysis, reinforcing that retroperitoneal bronchogenic cysts, though scarce, necessitate heightened clinical suspicion, particularly when patients present with nonspecific symptoms or incidental findings. Their study highlights the importance of considering bronchogenic cysts in the differential diagnosis of retroperitoneal masses. Therefore, it is necessary to have awareness of rare lesions and combine imaging and pathological results (3).

In addition, the results of imaging examinations were fully reflected in this study. CT and ultrasound provided preliminary information of lesion, and the final result depended on the pathology results (12). Some reports believed that the diagnostic rate of bronchogenic cysts was low, and many patients were misdiagnosed before surgery. In the present study, the imaging result displayed that the mass was a benign lesion, but the pathology result indicated that it was a bronchogenic cyst (9, 13, 14). Therefore, it is necessary to explore the lesion before surgery, and intraoperative exploration is very important because the surgeon should explore any abnormal findings cautiously (3).

This study discussed the occurrence of bronchogenic cysts in young females and was accompanied by bronchial developmental abnormalities. Most patients were male, and manifested typical symptoms in childhood and adolescence, while some patients presented as retroperitoneal masses, which might cause misdiagnosis with other benign lesions, such as lymphangiomas (4, 8). The results of imaging examinations provided information for surgery. However, the final result of the diagnosis depends on the pathology result. Literature reported that the accuracy of imaging was limited, and many patients were misdiagnosed before surgery (12). Therefore, the combination of imaging and pathology is very important to avoid misdiagnosis and missed diagnosis (15).

In addition, the importance of preoperative assessment and exploration during surgery was reflected in this study. Although the results of imaging provided information for surgery, exploration during surgery is very important to avoid any abnormal findings (3). Literature reported that accurate assessment before surgery and exploration during surgery can improve the prognosis of patients (16). In this case, the patient recovered well postoperatively, demonstrating the effectiveness of surgical treatment for retroperitoneal cystic masses. This also highlights the importance of postoperative follow-up to monitor recurrence risks and complications (12). In this study, the patient recovered well after surgery, and the prognosis was good. The surgical team can provide better diagnostic and therapeutic services for patients by exploring the cause together, especially in complicated cases (3).

The clinical discussion shows that the case of bronchogenic cysts is unique, and clinicians may misdiagnose it (17). This lesion is not very commonly found in young females; however, clinicians should be careful when faced with such cases. The imaging examination did not find the bronchogenic cyst well and delayed the initial diagnosis in this case. It is necessary to integrate imaging and pathology. Some studies have reported that the limitation of imaging decreased the diagnostic rate of bronchogenic cysts (18). Most patients were not diagnosed well before surgery, and the final diagnosis relies on pathology (19). Therefore, clinical practice should focus on integrating imaging features and clinical presentations.

The radiological appearance of bronchial cysts largely relies on the composition of their contents, including serous, mucinous, or proteinous content. Moreover, it can also impact the manifestations 2 mm, and of infection or bleeding; computed tomography (CT) is the desired technique to examine bronchial cysts. In general, the cysts are lesions presented with smooth edges, thin walls (less than 2 mm), and a homogenous density of the interiors, and the obtained CT attenuation is nearly identical to that of water (0–20 HU). Following enhancement scanning, mild enhancement in the cyst wall is observed, whereas the contents within the cyst lack any enhancement characteristic, which is a distinguishing facet when compared to hypervascular tumors or abscesses (20). Magnetic resonance imaging (MRI) has the merit of showing complex cysts, as well as evaluating their association with adjacent soft tissue. The signal intensity ranges between low (serous rate) and high (mucinous and rich in proteins). Almost all of the bronchial cysts seem to be seen as a bright signal due to a phenomenon known as the light bulb sign on T2-weighted images. DWI usually indicates that there is no limit to diffusion (21). Fluid density on CT grown beyond 20 HU appears when a cyst is either secondarily infected or hemorrhaging. The cyst wall also irregularly thickens and enhances significantly. These characteristics are easily confused and wrongly diagnosed as a necrotic tumor or abscess. Punctate or arc-shaped calcifications can be observed in the cyst wall in approximately 10–30 percent of cases, which, combined with being a benign finding, can be mistaken for other cystic lesions (22). Bronchial cysts are the most uncommon and most disorienting, and the incidence of them being out of the mediastinum, less than 1% of all instances, is not found in the retroperitoneum (23). This is a very rare scenario, however, this is the case. Its imaging features in this area are similar area-wise to those of lymphangiomas (usually multi-locular and infiltrative growth along fascial spaces), intestinal duplication cysts (in which the inner wall of the cyst can have gastrointestinal wall architecture such as muscle layers), or neurogenic cysts (24). Thus, although imaging results are highly positive for retroperitoneal unilocular cysts of thin-wall, the appearance of ectopic bronchial cysts must remain on the clinical list of diagnoses.

Histopathologic examination is the sole gold standard for diagnosing bronchial cysts because it enables the identification of characteristic tissue structures. This diagnosis is based on typical histological structures that represent their dysgenic appearance out of the embryonic bronchi that are in the form of a bud (25). The cysts in general appearance are grossly unilocular, thin, smooth-walled, with distal internally viscous or brownish-yellow fluid (old hemorrhage). The absolute need for microscopic diagnosis is the availability of ciliated pseudostratified columnar epithelium that lines the cyst wall, which is characteristic of respiratory epithelia. Furthermore, there is at least one bronchial-related structure that must be present in the wall of the cyst, i.e., islands of hyaline cartilage, smooth muscle bundles, or mucous/serous glands (25).

Using the literature framework, we re-evaluated the diagnosis and treatment of this case in order to understand it better. The case presented was a young girl who had a cystic mass in the retroperitoneal region. This presentation puts her on the general list of differential diagnoses. The main importance of imaging tests (CT and ultrasound) is to establish the fact that lesions are benign and cystic, excluding the possibility of malignancy, and hence dictating surgical care. Nevertheless, attempting to differentiate lymphangiomas and bronchial cysts with the help of imaging prior to surgery is hard to do. Finally, the lining of the ciliated columnar epithelium and glandular structures in the walls of the cyst were examined pathologically, and were able to lock in the diagnosis was confirmed. This is the very model of diagnosis that is localized and qualitatively assessed by means of imaging and eventually classifies the nature of pathology: this is the ideal representation of the optimal value of multidisciplinary diagnosis in contemporary precision medicine. The literature and this research prove that preoperative diagnosis of bronchial cysts, in particular, and ectopic cysts is very difficult, which means that it is crucial to create order in diagnostic procedures. Multimodal imaging (CT/MRI) is the initial approach to the diagnostic process, which is used to evaluate all features thoroughly and to detect atypical ones. Second, it should be an intraoperative, careful exploration, and cyst fluid should be aspirated and given for cytological analysis. The last and final one is to get complete pathological specimens, which will be examined under a microscope. A close association between the intraoperative decision-making of the surgeon and the microscopic diagnosis of the pathologist will help to obtain success in treating such problematic cases.

In summary, this case provides valuable experience in the clinical practice of bronchogenic cysts and lays the foundation for further research into the epidemiological characteristics and pathogenesis of this disease. This case report focused on the clinical significance of this rare lesion. There are still some limitations to this case report. Further research should analyze cases with larger sample sizes to further validate the effectiveness of integrating imaging and pathology and to study the pathogenesis of bronchogenic cysts in different populations. This will provide more guidance for clinicians facing similar cases and will help to improve clinical accuracy in diagnosis and treatment.

## Data Availability

The original contributions presented in the study are included in the article/supplementary material, further inquiries can be directed to the corresponding author.
